# Prevalence of vitamin D deficiency in women from southern Brazil and association with vitamin D-binding protein levels and *GC-DBP* gene polymorphisms

**DOI:** 10.1371/journal.pone.0226215

**Published:** 2019-12-12

**Authors:** Betânia Rodrigues Santos, Nathália Cruz Costa, Thais Rasia Silva, Karen Oppermann, Jose Antonio Magalhães, Gislaine Casanova, Poli Mara Spritzer

**Affiliations:** 1 Gynecological Endocrinology Unit, Division of Endocrinology, Hospital de Clínicas de Porto Alegre, Porto Alegre, Rio Grande do Sul, Brazil; 2 Laboratory of Molecular Endocrinology, Department of Physiology, Federal University of Rio Grande do Sul, Porto Alegre, Rio Grande do Sul, Brazil; 3 Medical School, Universidade de Passo Fundo and Hospital São Vicente de Paulo, Passo Fundo, Rio Grande do Sul, Brazil; 4 Division of Gynecology and Obstetrics, Hospital de Clínicas de Porto Alegre, Porto Alegre, Rio Grande do Sul, Brazil; Charles P. Darby Children's Research Institute, UNITED STATES

## Abstract

Vitamin D deficiency is highly prevalent worldwide, and vitamin D-binding protein (DBP) a major regulator of serum vitamin D levels. The rs4588 and rs7041 polymorphisms of the *GC* gene constitute the genetic basis of the three major isoforms of circulating DBP (GC1s, GC1f, and GC2), while the rs2282679 variant is located in an important regulatory region of the *GC* gene. The aim of this study was to assess the prevalence of 25-hydroxyvitamin D [25(OH)D] deficiency and to ascertain whether it is associated with DBP levels and with *GC* gene variants. Biorepository samples of 443 women aged 20 to 72 years, with no evidence of clinical disease, were analyzed. Circulating levels of 25(OH)D were considered sufficient if ≥20 ng/mL and deficient if <20 ng/mL. Genotype analysis was performed by RT-PCR. Mean age was 53.4±9.4 years; mean BMI was 27.8±5.8 kg/m^2^. The overall sample had mean 25(OH)D levels of 22.8±8.3 ng/mL; 39.7% of participants had deficient circulating 25(OH)D levels. Higher prevalence ratios (PR) of 25(OH)D deficiency were found for the CC genotype of rs2282679 (PR 1.74; 95%CI 1.30 to 2.24; p<0.001), GC2 isoform (PR 1.66; 95%CI 1.17 to 2.38; p = 0.005), time since menopause (PR 1.02; 95%CI 1.003 to 1.03, p = 0.016), and HOMA-IR (PR 1.02; 95%CI 1.01 to 1.03, p = 0.004). DBP levels (per 30 μg/mL increase in DBP) were associated with lower PR for 25(OH)D deficiency (PR 0.89; 95%CI 0.80;0.99; p = 0.027). Except for HOMA-IR, these prevalence ratios remained significant after adjustment for age and BMI. In conclusion, the rs2282679 polymorphism and the GC2 isoform of DBP were associated with lower serum DBP levels and with susceptibility to 25(OH)D deficiency in Brazilian women with no evidence of clinical disease.

## Introduction

Vitamin D deficiency is highly prevalent worldwide [1, 2] and has been regarded as a public health issue [3]. As a fat-soluble hormone, vitamin D is essential for the maintenance of calcium homeostasis, and has also been associated with hypertension, diabetes, the metabolic syndrome, cancer, autoimmune diseases, and infection, among other conditions [2, 4, 5].

Vitamin D-binding protein (DBP) is a member of the albumin and alpha-fetoprotein gene family encoded by the *GC* vitamin D binding protein gene (gene ID: 2638); it is produced in the liver, and its synthesis is regulated by estrogens. DBP is one of several regulators of serum vitamin D levels, with around 85–90% of the circulating vitamin D pool being bound to DBP [6, 7]. Total vitamin D correlates positively with DBP concentrations [8, 9].

Two well-studied single nucleotide polymorphisms (SNPs) of the *GC* gene, rs4588 and rs7041, form the molecular basis for the three major isoforms of circulating DBP (GC1f, GC1s, and GC2). These isoforms have different binding affinity for vitamin D [7] as well as different glycosylation patterns [10]. rs4588 and rs7041 have been associated with DBP concentrations [8, 11–14] and vitamin D levels [12, 15–18] in various populations. Another *GC* gene variant is the rs2282679 polymorphism, located at the 3’ untranslated region (3′ UTR) of the *GC* gene, which is involved in the modulation of gene expression and reportedly associated with vitamin D levels [19, 20].

Given this context, the aim of the present study was to assess circulating 25-hydroxyvitamin D [25(OH)D] and serum DBP levels and ascertain whether an association exists between the rs4588, rs7041, and rs2282679 polymorphisms of the *GC* gene and the presence of vitamin D deficiency in pre-, peri-, and postmenopausal women from southern Brazil.

## Materials and methods

### Study design and participants

This is a cross-sectional study of biorepository samples collected from 443 women aged 20 to 72 years, with no evidence of clinical disease, living in southern Brazil (30th parallel South). These women were prospectively recruited and participated in studies conducted at our research center from 2005 to 2012 [21–24]. Eighty percent of women were Caucasian and the remaining were of mixed African and European ancestry.

Serum samples had been previously collected and stored in aliquots at -80°C for use in laboratory tests. An additional blood sample was collected from each participant in EDTA tubes or on FTA Elute cards (GE Healthcare, Buckinghamshire, UK) for DNA extraction and polymorphism genotyping. Menopause status was ascertained based on the characteristics of menses or time since amenorrhea: premenopause was defined as usual menstrual frequency or flow; perimenopause was defined as changes in menstrual frequency; and postmenopause was defined as 12 or more months of amenorrhea and/or follicle-stimulating hormone (FSH) levels ≥35 mIU/mL occurring after 40 years of age [25]. Women who had undergone hysterectomy and/or bilateral oophorectomy were excluded. Circulating levels of 25(OH)D were considered sufficient if ≥20 ng/mL and deficient if *<*20 ng/mL. The study protocol was approved by the Ethics Commitee at Hospital de Clinicas de Porto Alegre (project 17–0226), and written informed consent was obtained from all subjects at the time of recruitment.

### Study protocol

As reported elsewhere [21–24], a physical examination was performed and blood pressure, weight, height, and waist circumference (WC) were measured. The body mass index (BMI) was calculated as weight in kg divided by the height in m squared (kg/m^2^). The metabolic syndrome and cutoff points for its isolated components were defined as per the Joint Scientific Statement [26]. Data on calcium and vitamin D supplementation and hormone therapy were collected at the time of recruitment.

### Laboratory parameters

All samples were obtained between 8:00AM and 10:00AM. Blood samples were drawn after a 12-h overnight fast for determination of laboratory analyses. Total cholesterol (TC), high-density lipoprotein cholesterol (HDL-c), and triglycerides were determined by enzymatic colorimetric methods (Bayer 1800 Advia System, Mannheim, Germany), with intra- and inter-assay coefficients of variation (CV) <3%. Low-density lipoprotein cholesterol (LDL-c) was calculated using the Friedewald formula [27]. The hexokinase method (Advia 1800, Mannheim, Germany) was used for glucose determination, with intra- and inter-assay CVs <3.4%. Serum insulin levels were measured by electrochemiluminescence immunoassay (ECLIA; Roche Diagnostics, Mannheim, Germany), with sensitivity of 0.200 μIU/mL and intra- and inter-assay CVs of 2.0% and 4.3% respectively. The homeostasis model assessment of insulin resistance index (HOMA-IR) was calculated by multiplying insulin (μIU/mL) by glucose (mmol/L) and dividing this product by 22.5 [28]. Estradiol was measured by electrochemiluminescence (Roche Diagnostics, Mannheim, Germany), with sensitivity of 5.0 pg/mL and intra- and inter-assay CVs of 5.7% and 6.4%, respectively. Individual results below the limit of sensitivity were considered equal to 5.0 pg/mL for purposes of statistical analysis. Levels of 25(OH)D were measured by chemiluminescence (Abbott Architect, IL, USA) with sensitivity of 1.6 ng/mL and intra- and inter-assay CVs of ≤5.1% and ≤7.1%, respectively. DBP was measured by commercial enzyme-linked immunosorbent assay (ELISA; R&D Systems, Minneapolis, USA), performed in accordance with the manufacturer’s instructions, with sensitivity of 0.180 ng/mL and intra- and inter-assay CVs of ≤2.2% and ≤6.5%, respectively. Serum albumin was measured using an automated colorimetric method (Vitros, São Paulo, Brazil). Free and bioavailable 25(OH)D levels were calculated using equations provided by Powe et al. [29], which in turn were adapted from the equations developed by Vermeulen et al. for calculation of free testosterone [30] and based on the concentration of total testosterone, sex hormone-binding globulin (SHBG), and albumin. The affinity binding constants for 25(OH)D with DBP and albumin were 7×10^8^ M^−1^ and 6×10^5^ M^−1^, respectively, as previously measured by Bikle et al. by centrifugal ultrafiltration dialysis [31]. Parathyroid hormone (PTH) and calcium levels were measured by chemiluminescent microparticle immunoassay (CMIA) (Abbott Architect, Wiesbaden, Germany) and the O-cresolphthalein colorimetric (Advia 1800, Mannheim, Germany) method respectively.

### Genotype analysis

Genomic DNA was extracted from peripheral blood leukocytes [32] or from the FTA Elute cards according to the manufacturer’s protocol (GE Healthcare). DNA samples were diluted to 2 ng/mL. Duplicate measurements were performed in 10% of the samples to assess the internal quality of genotype data. Molecular genotyping for rs4588 (substitution of C for A), rs7041 (substitution of T for G), and rs2282679 (substitution of A for C) was performed through real-time polymerase chain reaction (PCR) (ViiA7 Real-Time Polymerase Chain Reaction System, Applied Biosystems, California, USA) using the allelic discrimination assay with TaqMan MGB primers and probes (Applied Biosystems, California, USA). BDP isoforms were constructed from the combination of the rs4588 and rs7041 polymorphisms, formally called GC1s (rs4588 CC and rs7041 GG), GC1f (rs4588 CC and rs7041 TT), and GC2 (rs4588 AA and rs7041 TT).

### Statistical analysis

The Shapiro-Wilk normality test and descriptive statistics were used to evaluate the distribution of data. Results are presented as mean ± standard deviation (SD), median and interquartile range, or percentage. Non-Gaussian variables were log-transformed for statistical analysis and reported after being back-transformed into their original units of measurement. One-way analysis of variance (ANOVA) was used to compare group means. A test for linear trend was used to detect codominant effects of genotypes on DBP and 25(OH)D levels. Pearson’s chi-square test (χ^2^) was used to test categorical variables and the agreement of genotype frequencies with Hardy-Weinberg equilibrium. The relation of the outcome of interest (25(OH)D status) with genotypes, DBP isoforms, DBP levels, time since menopause, HOMA-IR, and estradiol was evaluated using prevalence ratios (PR) estimated by univariate Poisson regression with robust variance. A multivariate Poisson regression model with robust variance was constructed to adjust the analysis for age and BMI (model 1) and model 1 plus vitamin D supplementation (model 2). The 25(OH)D ≥20 ng/mL category was used as reference. All analyses were performed in PASW Statistics for Windows, Version 18.0 (SPSS Inc., Chicago, IL, USA). Significance was accepted at p<0.05.

## Results

Table 1 summarizes the clinical and biochemical profile of the 443 participants according to menopause status (13.8% premenopausal, 5.2% perimenopausal, and 81.0% postmenopausal). The peri- and postmenopausal women had significantly higher blood pressure, TC, LDL-c, and HOMA-IR, as well as lower estradiol and DBP levels, than premenopausal participants (p<0.05 for all variables). The mean serum 25(OH)D level in the overall sample was 22.80±8.32 ng/mL; 60.3% had sufficient circulating 25(OH)D levels (≥20 ng/mL), while 39.7% had deficient 25(OH)D levels (*<*20 ng/mL). The three subgroups had similar prevalence of vitamin D deficiency and circulating 25(OH)D levels (Table 1). Participants were mostly Caucasian (80%); 20% were of mixed African and European ancestry. Overall, 8.9% and 5.9% of participants were taking calcium and vitamin D supplementation, respectively, and 7.9% were on hormone therapy. PTH and calcium levels measured in a subset of 84 postmenopausal women were within the reference range [median PTH: 41.75 pg/mL (32.83–50.75); mean calcium: 9.10±0.33 mg/dL].

**Table 1:** Clinical and biochemical profile of participants according to menopause status.

Variable	Premenopause (n = 61)	Perimenopause (n = 23)	Postmenopause (n = 359)	p
Age (years)	36.84±11.17^a^	51.83±2.71^b^	56.36±5.61^c^	<0.001
BMI (kg/m^2^)	28.74±7.86	28.62±5.40	27.63±5.42	0.315
WC (cm)	86.43±13.42	89.83±11.91	88.67±12.58	0.391
Systolic BP (mmHg)	119.70±15.01^a^	130.09±14.89^ab^	128.55±18.33^b^	0.002
Diastolic BP (mmHg)	78.82±11.44^a^	86.61±13.59^b^	81.58±11.50^ab^	0.026
Glu (mg/dL)	88.75±10.96	92.00±16.82	95.00±25.59	0.160
TC (mg/dL)	185.31±35.69^a^	204.30±33.27^ab^	212.84±41.16^b^	<0.001
HDL-c (mg/dL)	51.55±11.67	52.65±11.46	55.39±14.17	0.109
Triglycerides (mg/dL)	114.3 (92.8–131.3)	121.0 (75.0–222.0)	124.0 (88.0–167.8)	0.385
LDL-c (mg/dL)	102.67±52.38^a^	120.75±38.01^ab^	129.02±34.21^b^	<0.001
HOMA-IR	2.14 (1.31–2.90)^a^	1.81 (1.04–3.18)^ab^	1.81 (1.24–2.78)^b^	0.042
Estradiol (pg/mL)	53.7 (24.3–99.0)^a^	6.8 (5.0–89.5)^b^	7.0 (5.0–18.3)^c^	<0.001
Metabolic syndrome				
No	44 (78.6%)	12 (54.5%)	239 (67.5%)	0.093^†^
Yes	12 (21.4%)	10 (45.5%)	115 (32.5%)	
25(OH)D (ng/mL)	21.69±7.39	22.16±7.94	23.04±8.49	0.471
<20 ng/mL	28 (45.9%)	07 (30.4%)	141 (39.3%)	0.400^†^
≥20 ng/mL	33 (54.1%)	16 (69.6%)	218 (60.7%)	
DBP (μg/mL)	214.63±28.08^a^	183.81±36.31^b^	196.46±29.50^b^	<0.001
Free 25(OH)D (pg/mL)	7.36±2.77	8.30±3.28	8.40±3.41	0.090
Bioavailable 25(OH)D (ng/mL)	3.26±1.25	3.85±1.56	3.66±1.54	0.133

Data are expressed as mean ± SD, median (interquartile range) or percentage. p value by one-way ANOVA (different superscript letters indicate statistical difference between groups) or Pearson’s χ^2^ test (^†^). BMI: body mass index; WC: waist circumference; BP: blood pressure; Glu: glucose; TC: total cholesterol; HDL-c: high-density lipoprotein cholesterol; LDL-c: low-density lipoprotein cholesterol; HOMA-IR: homeostasis model assessment index to estimate insulin resistance; 25(OH)D: 25-hydroxyvitamin D; DBP: vitamin D-binding protein. Missing values of metabolic syndrome:11.

Genotype and DBP isoform frequencies of *GC* gene variants are shown in Table 2. Only two participants were not genotyped for SNP rs4588, and three for both SNP rs7041 and rs2282679. All three SNPs were in Hardy-Weinberg equilibrium (rs4588: χ^2^ = 1.47, p = 0.23; rs7041: χ^2^ = 2.88, p = 0.09; rs2282679: χ^2^ = 0.17, p = 0.68).

**Table 2:** *GC* gene polymorphisms and DBP isoforms frequencies.

SNP	n (%)
rs4588	
CC	226 (51.2%)
CA	172 (39.0%)
AA	43 (9.8%)
rs7041	
TT	126 (28.6%)
TG	202 (45.9%)
GG	112 (25.5%)
rs2282679	
AA	228 (51.8%)
AC	175 (39.8%)
CC	37 (8.4%)
DBP isoforms	
GC1s	110 (60.8%)
GC1f	33 (18.2%)
GC2	38 (21.0%)

rs4588: 2 missing; rs7041 and rs2282679: 3 missing. GC1s (rs4588 = CC and rs7041 = GG); GC1f (rs4588 = CC and rs7041 = TT); GC2 (rs4588 = AA and rs7041 = TT).

**Fig 1:**
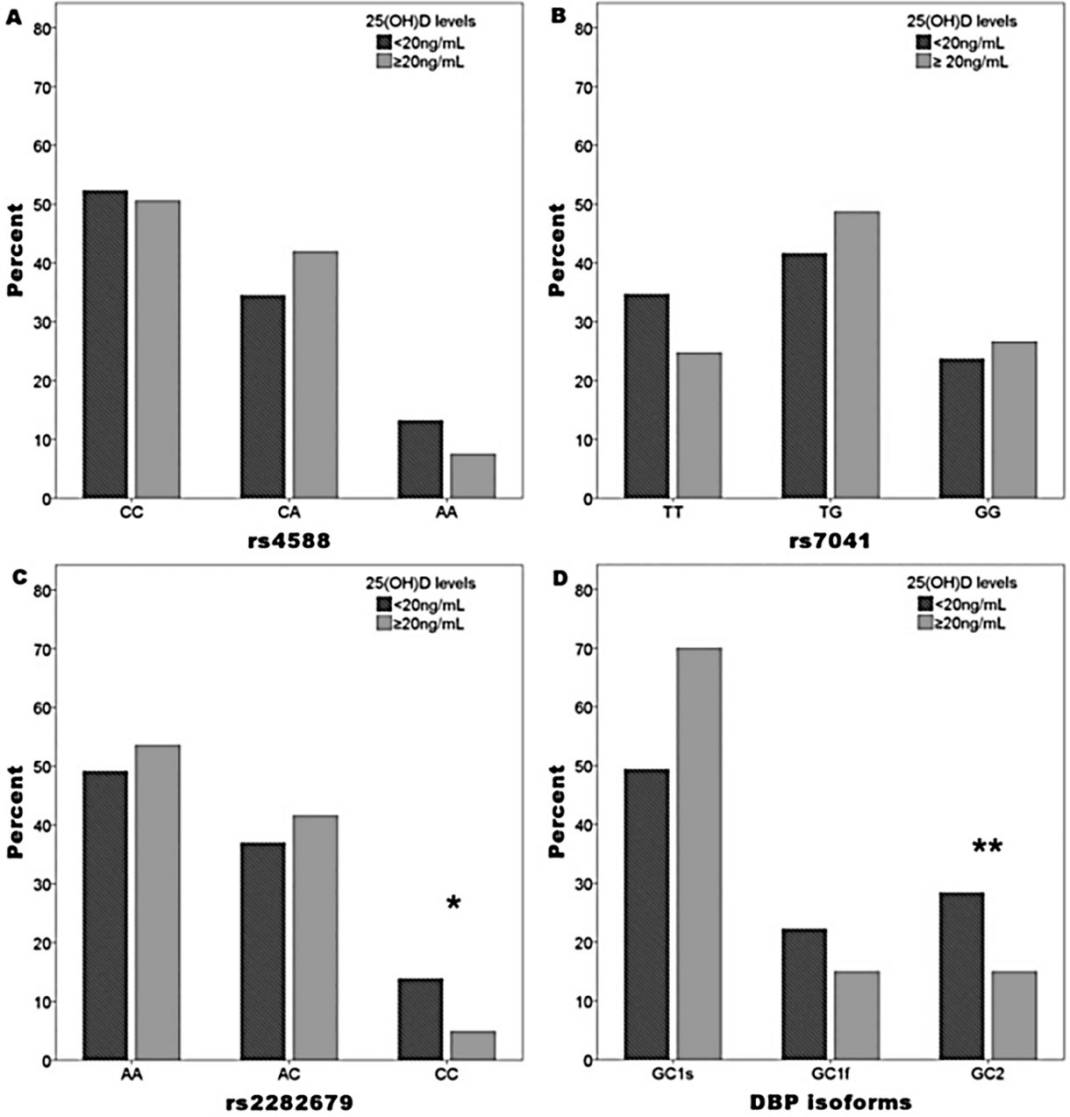
*GC* gene genotype and DBP isoform distribution according to 25(OH)D status. Data are expressed as percentages; p value by Pearson’s chi-square test. *p = 0.004 for SNP rs2282679; **p = 0.016 for GC2 DBP isoform.

Regarding the distribution of *CG* gene variants according to vitamin D *status*, CC genotype frequencies of rs2282679 and GC2 DBP isoform were significantly higher in participants with 25(OH)D <20 ng/mL than in participants with ≥20 ng/mL (13.9% vs. 4.9%, p = 0.004; and 28.4% vs. 15.0%, p = 0.016 respectively) (Fig 1).

Table 3 shows DBP and 25(OH)D serum levels according to genotypes of *GC* gene polymorphisms and DBP isoforms. The AA genotype of rs4588, CC genotype of SNP rs2282679, and the GC2 isoforms were associated with lower DBP, but no such association was found for SNP rs7041. The TT genotype of rs7041, CC genotype of rs2282689, and DBP GC2 isoform were also associated with lower 25(OH)D levels.

**Table 3:** DBP and 25(OH)D levels according to *GC* gene genotype and DBP isoforms.

SNP	DBP (μg/mL)	B	Linear p trend	25(OH)D (ng/mL)	B	Linear p trend
rs4588						
CC	202.98±28.28	-8.4	<0.001	23.00±8.84	-0.6	0.282
CA	196.49±29.88			23.16±7.83		
AA	183.95±36.85			20.77±7.15		
rs7041						
TT	192.96±33.41	3.5	0.078	21.48±7.54	1.2	0.030
TG	201.38±28.06			23.20±8.24		
GG	199.82±30.02			23.78±9.14		
rs2282679						
AA	203.13±27.90	-9.3	<0.001	23.39±8.79	-1.3	0.034
AC	196.41±30.04			22.83±7.80		
CC	180.88±38.20			19.70±7.17		
DBP isoforms						
GC1s	199.41±29.89	-7.7	0.012	23.83±9.19	-1.9	0.015
GC1f	204.83±31.58			20.63±8.29		
GC2	180.75±37.58			20.25±7.39		

Data are expressed as mean ± SD. p value by linear trend test.

Prevalence ratios for 25(OH)D <20 ng/mL according to rs2282679 genotype, DBP isoforms, DBP levels, time since menopause, HOMA-IR and estradiol are shown in Table 4. A higher risk of 25(OH)D <20 ng/mL was associated with CC genotype of rs2282679 (PR 1.740; 95%CI 1.301 to 2.237; p<0.001), GC2 isoform (PR 1.664; 95%CI 1.165 to 2.377; p = 0.005), time since menopause (PR 1.019; 95%CI 1.003 to 1.034; p = 0.016) and HOMA-IR (PR 1.018; 95%CI 1.006 to 1.033; p = 0.004). A lower risk of 25(OH)D <20 ng/mL was associated with DBP levels (per 30 μg/mL increase in DBP: PR 0.886; 95%CI 0.796 to 0.987; p = 0.027). These prevalence ratios remained significant even after adjustment for age, BMI, and vitamin D supplementation.

**Table 4:** Prevalence ratios for 25(OH)D<20ng/mL according to rs2282679 genotypes, DBP isoforms, DBP levels, time since menopause, HOMA-IR, and estradiol levels.

Variable	PR (95% CI)	p value	PR (95% CI)^1^	p value^1^	PR (95% CI)^2^	P value^2^
rs2282679						
AA	1.0	-	1.0	-	1.0	-
AC	0.981 (0.758–1.269)	0.884	0.978 (0.753–1.269)	0.865	0.963 (0.740–1.254)	0.780
CC	1.740 (1.301–2.237)	<0.001	1.738 (1.303–2.318)	<0.001	1.696 (1.263–2.276)	<0.001
DBP isoforms						
GC1s	1.0	-	1.0	-	1.0	-
GC1f	1.500 (1.008–2.232)	0.046	1.473 (0.978–2.218)	0.064	1.486 (0.993–2.223)	0.054
GC2	1.664 (1.165–2.377)	0.005	1.626 (1.147–2.306)	0.006	1.593 (1.120–2.265)	0.010
DBP (per 30 μg/mL increase)	0.886 (0.796–0.987)	0.027	0.876 (0.787–0.975)	0.015	0.881 (0.790–0.982)	0.022
Time since menopause (years)	1.019 (1.003–1.034)	0.016	1.032 (1.012–1.053)	0.002	1.033 (1.012–1.054)	0.001
HOMA-IR	1.018 (1.006–1.033)	0.004	1.015 (0.999–1.030)	0.063	1.015 (1.000–1.030)	0.046
Estradiol (pg/mL)	1.001 (0.999–1.003)	0.332	1.001 (0.999–1.004)	0.405	1.001 (0.999–1.004)	0.394

p value by univariate Poisson regression with robust variance. ^1^model adjusted for age and BMI; ^2^model adjusted for age, BMI, and vitamin D supplementation. DBP: vitamin D-binding protein; HOMA-IR: homeostasis model assessment index to estimate insulin resistance.

## Discussion

In the present study, the CC genotype of the rs2282679 SNP and the GC2 isoform of the *GC* gene were associated with lower DBP and total 25(OH)D levels, as well as with higher risk of vitamin D deficiency. Moreover, the prevalence ratio of vitamin D deficiency was almost twice as high in carriers of these gene variants.

Few studies have assessed the impact of GC-DBP gene polymorphisms on 25(OH)D levels or measured serum DBP concentrations in women of diverse ethnic origins at different reproductive periods. Indeed, previous studies in other women populations have shown discrepant results–a lack of association [33–35] in some cases, or conversely a relationship between *GC* gene polymorphisms and 25(OH)D levels [8, 11, 12, 15, 16, 36, 37]. In addition, an association of these gene with 25(OH)D levels has been observed in specific conditions, such as polycystic ovary syndrome (PCOS) [17], type 2 diabetes [18], and early breast cancer [38]. Beyond that, previous studies have reported an association between SNP rs2282679 and vitamin D deficiency [19, 20], and between DBP levels and its gene polymorphisms [8, 11–14], except for one study with negative results [34]. Our finding of an association between the GC2 isoform and lower DBP levels in southern Brazilian women is in line with these previous finings in other populations.

Interestingly, the distribution of DBP isoforms has been reported to vary according to distinct ethnic patterns. Black and Asian populations are more likely to carry the GC1f isoform, and only rarely the GC2, whereas whites more frequently exhibit the GC1s and GC2 isoforms [6, 7]. The present results are consistent with this observation, since our participants, who were mostly of white European descent, had a higher frequency of the GC1s isoform.

We found that DBP levels were lower in peri- and postmenopausal women than in premenopausal women. DBP synthesis in the liver is regulated by sex-steroid hormones, particularly estrogen, which stimulates hepatic DBP synthesis. Thus, during the menopausal transition and postmenopause, changes in ovarian estrogen secretion may account for lower DBP and total vitamin D circulating concentrations [6, 9]. Although our data in this sample of women with no clinical evidence of disease did not show differences in total, free, or bioavailable 25(OH)D levels between groups according to menopause status, the time since menopause was associated with a higher prevalence ratio, and DBP levels with a lower prevalence ratio, of 25(OH)D deficiency, independently of age and BMI. These data support emerging evidence regarding a positive correlation between total 25(OH)D concentrations and DBP levels [8, 9]. Indeed, around 85–90% of the circulating vitamin D pool are bound to DBP, especially the 25(OH)D form, which has higher binding affinity than 1,25-dihydroxyvitamin D [1,25(OH)_2_D] [6, 7].

In addition, we found a relationship between HOMA-IR and higher odds of vitamin D deficiency, even after adjustment for age, BMI and vitamin D supplementation. In fact, insulin resistance has been associated with lower circulating levels of vitamin D in women at different reproductive stages, such as after the menarche [39], during reproductive years [40], and in the pre- [41] and postmenopause [42]. In women with PCOS, who usually have an unfavorable metabolic profile, vitamin D levels were inversely associated with insulin resistance (measured by clamp or HOMA-IR) [43] and with metabolic syndrome and higher glucose and triglycerides levels [5]. A recent review of *in vitro*, animal, and human *in vivo* studies also underlines the association between vitamin D deficiency and cardio-metabolic variables related to insulin resistance [44]. A few studies have also reported that genetic variations in *GC-DBP gene* were associated with insulin resistance and normal glucose tolerance in Japanese [45] and with metabolic syndrome in PCOS women [17].

The strengths of our study include novel data on Brazilian women, a less well-represented population in studies about determinant factors of vitamin D levels. Furthermore, the sample included participants with no evidence of clinical disease, which reduces the possible interference of pathological processes in our findings. Limitations of this study include the lack of data on dietary vitamin D intake and daily sun exposure, even though it is well recognized that, at latitudes below 35°, UVB radiation is sufficient for year-round vitamin D synthesis [46].

## Conclusion

Data from this study suggest that the CC genotype of rs2282679 and the GC2 isoform of DBP are related to lower serum DBP levels and with susceptibility to 25(OH)D deficiency in adult and postmenopausal women with no evidence of clinical disease, independently of age and BMI.
